# Bayesian Age-Period-Cohort Prediction of Mortality of Type 2 Diabetic Kidney Disease in China: A Modeling Study

**DOI:** 10.3389/fendo.2021.767263

**Published:** 2021-10-29

**Authors:** Xiaoming Wu, Jianqiang Du, Linchang Li, Wangnan Cao, Shengzhi Sun

**Affiliations:** ^1^The Key Laboratory of Biomedical Information Engineering of Ministry of Education, School of Life Science and Technology, Xi’an Jiaotong University, Xi’an, China; ^2^Department of Clinical Medicine, Second Clinical School of Medicine, Shaanxi University of Chinese Medicine, Xianyang, China; ^3^Center for Evidence Synthesis in Health, Brown University School of Public Health, Providence, RI, United States; ^4^Department of Environmental Health, Boston University School of Public Health, Boston, MA, United States

**Keywords:** type 2 diabetic kidney disease, mortality, age-period-cohort modeling, projection, demographic change

## Abstract

**Background:**

The burden of type 2 diabetic kidney disease (DKD) continues to rise in China. We analyzed time trends in DKD mortality and associations with age, period, and birth cohort from 1990 to 2019, made projections up to 2030, and examined the drivers of deaths from DKD.

**Methods and Findings:**

The number of DKD deaths in China from 1990 to 2019 was obtained from the GBD 2019. We used age-period-cohort modeling to estimate age, period, and cohort effects in DKD mortality between 1990 and 2019. We calculated net drift (overall annual percentage change), local drift (annual percentage change in each age group), longitudinal age curves (expected longitudinal age-specific rates), period, and cohort relative risks. We used Bayesian age-period-cohort analysis with integrated nested Laplace approximations to project future age-specific DKD death cases from 2020 to 2030. We used a validated decomposition algorithm to attribute changes in DKD deaths to population growth, population aging, and epidemiologic changes from 1990 to 2030. From 1990 to 2019, the age-standardized mortality rate of DKD in China was relatively stable, but the absolute number of DKD deaths showed a noticeable increasing trend. The overall annual percentage change (net drift) was -0.75% (95% confidence interval, CI: -0.93 to -0.57) for males and -1.90% (95% CI, -2.19 to -1.62) for females. The age-specific annual percentage changes (local drifts) were below zero in all age groups from 1990 to 2019 except for males aged above 65 to 69 years, and for females aged above 70 to 74 years. The risk of DKD deaths increased exponentially with age for both sexes after controlling for period deviations. The Bayesian age-period-cohort analysis projects that there would be 88,803 deaths from DKD in 2030, increased by 224.2% from 1990. Despite a decrease in age-specific DKD death rates, the reduction would be entirely offset by population aging.

**Conclusions:**

Although China has made progress in reducing DKD deaths, demographic changes have entirely offset the progress. The burden of DKD deaths is likely to continue increasing. Our findings suggest that large-scale screening is imperative for DKD control and prevention, particularly for high-risk groups.

## Introduction

Type 2 diabetic kidney disease (DKD) is a common microvascular complication of type 2 diabetes mellitus (T2DM), occurs in approximately 20%-30% of diabetic patients, and is one of the leading causes of end-stage renal disease (ESRD) (1–3). DKD manifests as albuminuria, impaired glomerular filtration rate (GFR), or both, and even mild albuminuria and reduced GFR are associated with a significantly increased risk of cardiovascular disease and death (4, 5). In addition, patients with DKD-ESRD have a high mortality rate than non-DKD ESRD patients (6). Epidemiological studies have suggested that DKD has become the leading cause of chronic kidney disease (CKD) in the pre-dialysis CKD population in China, surpassing glomerulonephritis, and therefore will become the leading cause accounting for dialysis in the near future (7). The prevalence of DKD increases in direct proportion to the prevalence of T2DM (8); thus, the burden of DKD in China is likely to continue to increase as the prevalence of T2DM has risen sharply (7).

Although there has been an increasing trend in DKD burden in China across time (7, 9–11), the approaches used in previous studies fail to differentiate the relative contribution of period and cohort effects to overall time trends, which hinders us from evaluating the success of earlier policy interventions. We aimed to address this knowledge gap by evaluating how age, calendar period, and birth cohort are associated with increased mortality from DKD in China using an age-period-cohort analysis. Age effects are the changes related to the biological and social processes of aging specific to an individual. Period effects are caused by external factors that affect all age groups within a given calendar time. Cohort effects result from the unique experience or exposure of a group of subjects (the cohort) at different times (12, 13).

Estimation of future DKD mortality trends is vital for DKD control planning. We used Bayesian age-period-cohort analysis to predict future DKD deaths, which has been extensively used to predict the future burden of many diseases (14, 15). To analyze the drivers of DKD deaths, we used a validated decomposition algorithm (16) to attribute changes in the number of DKD deaths to population growth, population aging, and epidemiological changes in DKD. The findings of this study will improve our understanding of the time trends of DKD burden in China and identify potential drivers for the changes in DKD deaths, which may help guide public health policy, resource allocation, and the design of screening programs.

## Methods

### Study Data

We obtained China DKD mortality data from the Global Burden of Disease (GBD) Study 2019, which is a multinational collaborative study that estimates disease burden in 204 countries and territories worldwide (17, 18). The methods used in GBD 2019 have been reported in detail elsewhere (17–19). In brief, GBD 2019 used vital registration and verbal autopsy data to model mortality due to chronic kidney disease (CKD) (17). The Bayesian geospatial regression model was used to increase the comparability of mortality data sources that used location-specific covariates to create smoothed time trends. Data from the ESRD registry were used to estimate five causes of CKD: type 1 diabetes, type 2 diabetes, glomerulonephritis, hypertension, and a residual category of other and unspecified causes. The DKD data analyzed in this paper refer to the data on CKD due to type 2 diabetes. An epidemiologic state-transition disease modeling tool was used to produce consistent estimates by location, year, age, and sex. These adjusted proportions were applied to the parent CKD regression model to obtain type-specific estimates of CKD. As the data were publicly available and data were aggregated and de-identifiable, institutional review board approval and informed consent were not needed.

### Statistical Analysis

We used the age-period-cohort framework to estimate the following parameters: (1) net drift, representing the overall log-linear trend by period and birth cohort, indicating the overall annual percentage change of the expected age-adjusted rate; (2) local drifts, representing the log-linear trends for each age group by period and birth cohort, indicating the annual percentage change of the expected age-specific rate over time; (3) longitudinal age curve, showing the expected age-specific rates adjusted for period effects in reference cohort; (4) period (or cohort) rate ratios (RR), representing the ratio of age-specific rates in each period (or cohort) relative to the reference one.

For age-period-cohort analyses, we arranged the DKD mortality and population data into consecutive 5-year periods from 1990 to 2019, and successive 5-year age intervals from 15-19 years to 95 plus. The birth cohort was defined using the difference between the medium value of the age interval and the period interval. We obtained the estimated parameters by the age-period-cohort Web Tool provided by the National Cancer Institute (20). For relative rate measurements, the reference period interval was from 2000 to 2004, and the reference birth cohort interval was from 1945 to 1949. We used the Wald chi-square test to test the significance of the estimable parameters and functions. All statistical tests were two-sided.

We used the Bayesian age-period-cohort analysis with integrated nested Laplace approximations to project the future age-specific number of death cases from DKD from 2020 to 2030 (21), which shows better coverage and precision than other prediction methods (22). Based on the assumption that the effects of age, period and cohort adjacent in time are similar, the Bayesian inference in age-period-cohort model applies the second-order random walk for smoothing priors of age, period, and cohort effects and to project posterior mortality rates. The integrated nested Laplace approximations are used with this Bayesian age-period-cohort model to approximate the marginal posterior distributions avoiding any mixing and convergence issues introduced by Markov chain Monte Carlo sampling techniques traditionally used in the Bayesian approach. We conducted the Bayesian age-period-cohort analysis using R-package BAPC (version 0.0.34). We provided additional details in **Text S1**. The population predictions for China were taken from the 2019 revision of the United Nations (UN) World Population Prospects and were used to estimate China’s population in 2020 and beyond (23).

To analyze the drivers of the changes in the number of DKD deaths from 1990 to 2030, we used a newly developed decomposition method to attribute changes in the total number of DKD deaths to population growth, population aging, and age-specific changes in DKD mortality between 1990 and each subsequent year from 1991 to 2030 (16, 24). Briefly, this decomposition method has considered the 2-way and 3-way interactions of the three components and is robust to the choice of the decomposition order of the three factors, and the selection of the reference year compared to previous decomposition methods (25, 26). Details about the decomposition method were described elsewhere (16, 24) and in the **Text S2**. This method has been used to quantify the impact of population aging on mortality for 195 countries or territories and 169 causes of deaths (24), and to quantify the demographic and epidemiologic drivers for the impacts of air pollution and high sodium intake (27–29). We calculated the absolute and relative contributions of the three drivers to the change in the number of DKD deaths. The absolute contribution was the number of attributed DKD deaths, while the relative contribution was estimated as the number of attributed DKD deaths divided by the total DKD deaths in 1990×100%. A positive contribution indicates an increase in total DKD deaths, while a negative contribution indicates a decrease in total DKD deaths. The age-specific changes in DKD deaths refer to epidemiologic changes, which include all differences in mortality that cannot be explained by population growth and population aging (30), such as new treatments or medications for DKD. The net changes in these three components are equal to the difference in the total number of observed deaths. We performed statistical analyses with R software (Version 3.6.3, R core team).

## Results

### Trends in DKD Mortality

**Figure 1:**
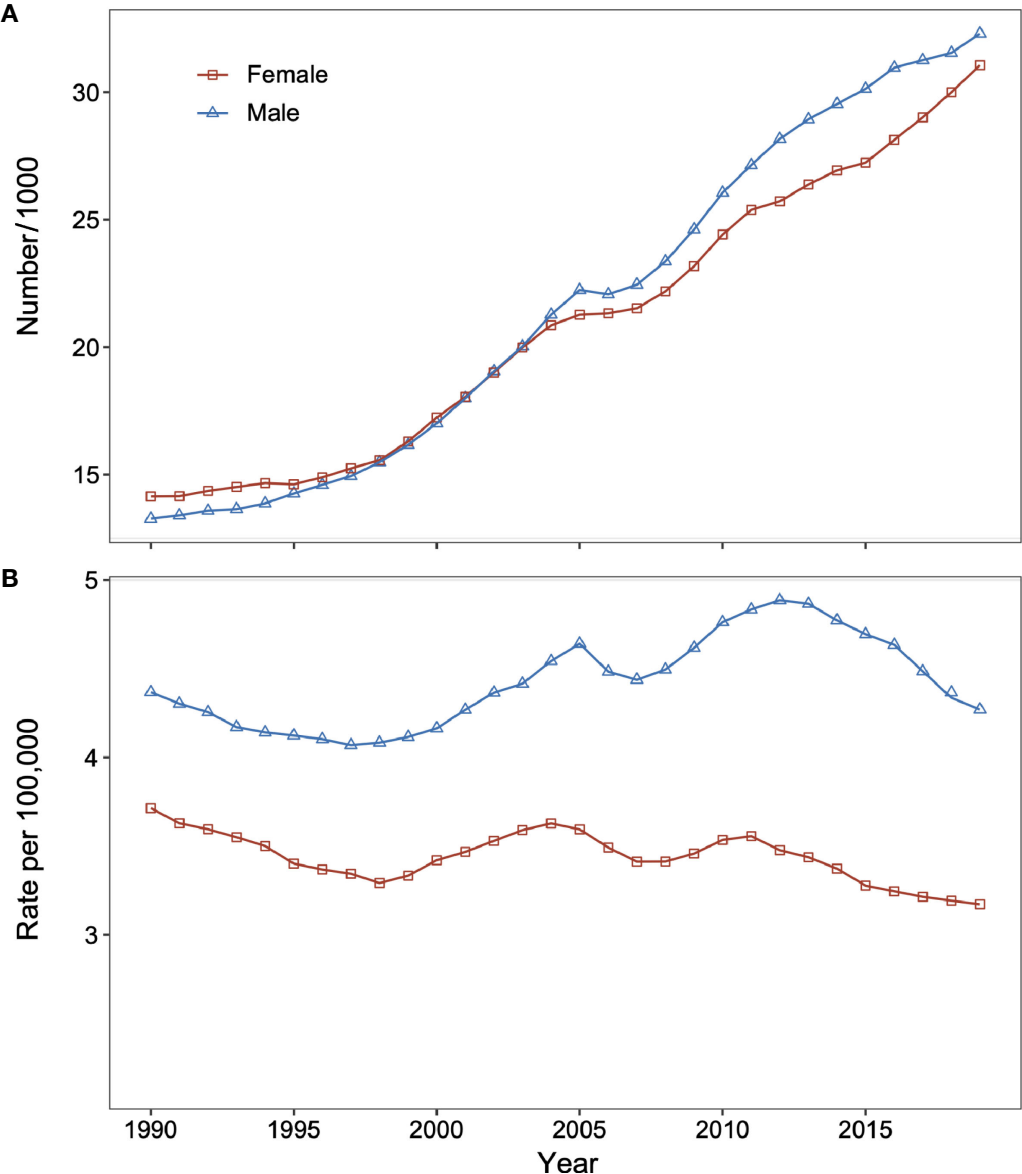
Changes in type 2 diabetic kidney disease (DKD) mortality and number of deaths for males and females in China from 1990 to 2019. **(A)** Number of DKD deaths for males and females. **(B)** Age-standardized death rate of DKD for males and females. DKD, type 2 diabetic kidney disease.

In 2019, there were 63,354 (95% UI: 49,787 to 77,280) DKD deaths in China, and the age-standardized mortality rate of DKD was 3.6 (95% UI: 2.8 to 4.3) per 100,000. Between 1990 and 2019, the total number of DKD deaths increased dramatically from 13,269 (95% UI: 9,998 to 16,930) in 1990 to 32,296 (95% UI: 24,275 to 41,393) in 2019 for males and from 14,144 (95% UI: 10,909 to 17,470) in 1990 to 31,058 (95% UI: 23,720 to 38,907) in 2019 for females (**Figure 1A**). On the contrary, the age-standardized mortality rate of DKD was relatively stable for both sexes (**Figure 1B**).

### Age-Period-Cohort Analysis

**Figure 2:**
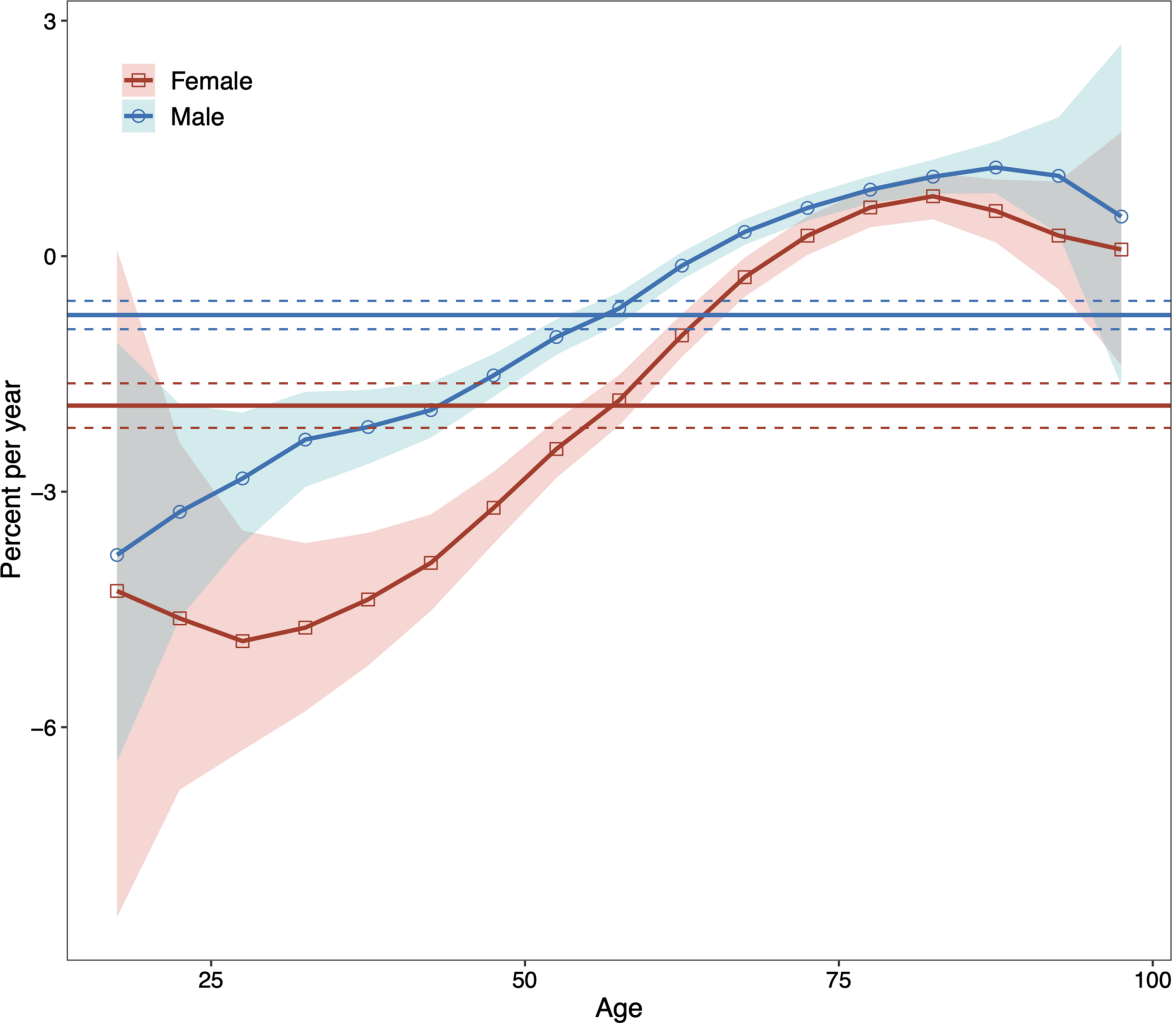
Local drifts with net drift values for males and females for type 2 diabetic kidney disease (DKD) mortality in China from 1990 to 2019. The horizontal solid lines are the net drifts, and the dashed lines showed their 95% confidence intervals. The solid line of the curve are the local drifts and the shaded area indicate their 95% confidence intervals. DKD, type 2 diabetic kidney disease.

Net drift represents the overall annual percentage change across the study period (**Figure 2**). We found marked sex differences in net drift with -0.75% (95% confidence interval, CI: -0.93% to -0.57%) for males and -1.90% (95% CI: -2.19% to -1.62%) for females, reflecting less improvement in reduction of DKD mortality for males than for females from 1990 to 2019. Local drift reflects additional age-specific variations in DKD mortality trends (**Figure 2**). Values lie predominantly below 0 for both sexes for most age groups, indicating improvements in reducing DKD mortality. The exceptions were males aged above 65 to 69 and females aged above 70 to 74, indicating increased mortality from DKD.

**Figure 3:**
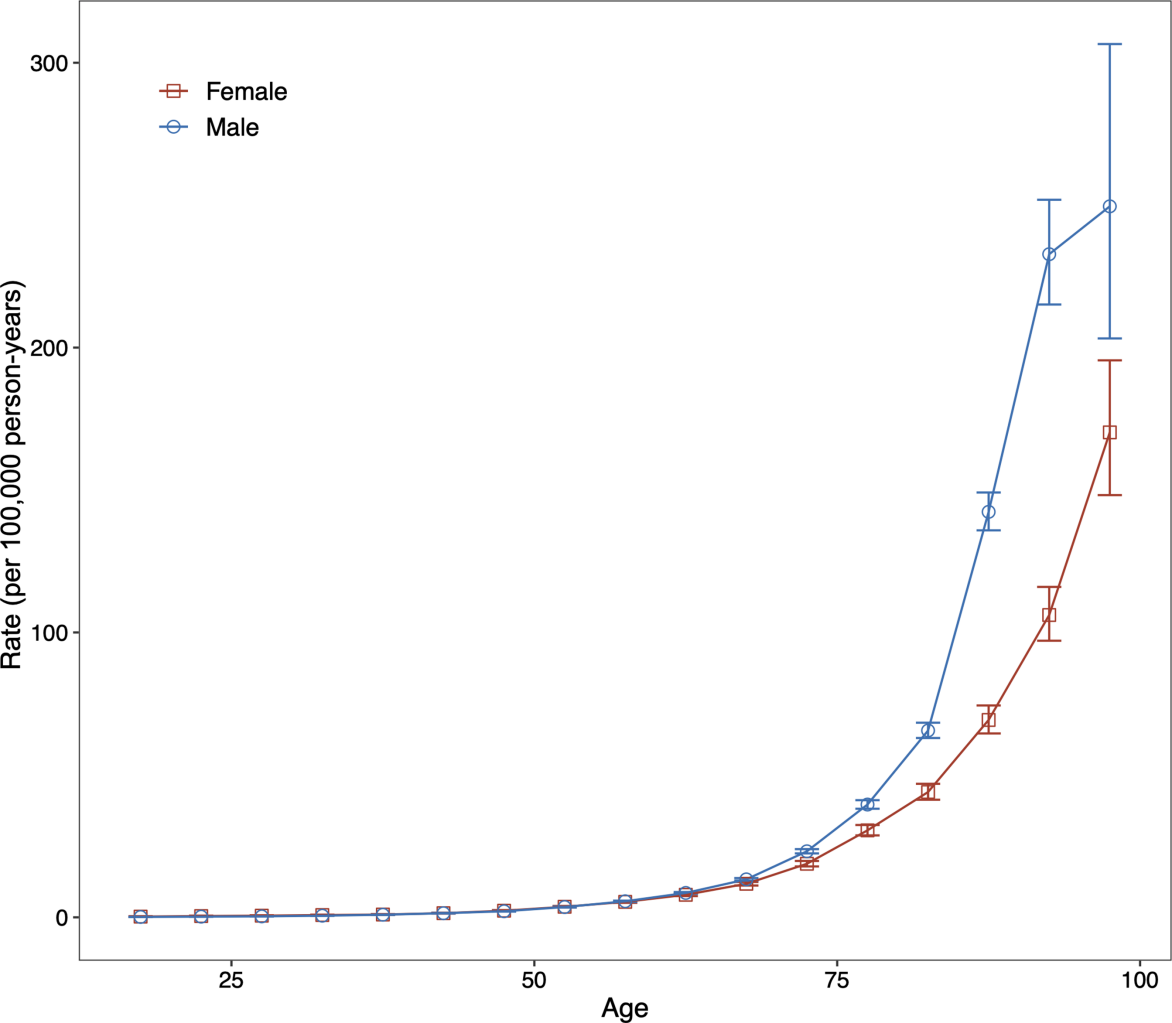
Fitted longitudinal age curves of type 2 diabetic kidney disease (DKD) mortality (per 100,000 person-years) and the corresponding 95% confidence interval for males and females. DKD, type 2 diabetic kidney disease.

For both sexes, in the same birth cohort, the risk of death from DKD showed an accelerated increase with age. We performed a curve estimation for the longitudinal age curves and found that both sexes showed an exponential distribution (**Figure 3**). The relationship between age and mortality rate can be expressed as mortality rate=0.023×e^0.097×age^ for males (R-squared=0.997) and mortality rate=0.056×e^0.081×age^ for females (R-squared=0.997), where age is the median age of each age interval. These indicated that the mortality risk of DKD was 128-fold higher for males and 57-fold higher for females aged 75 to 79 years compared to the corresponding males and females aged 20 to 24, respectively.

**Figure 4:**
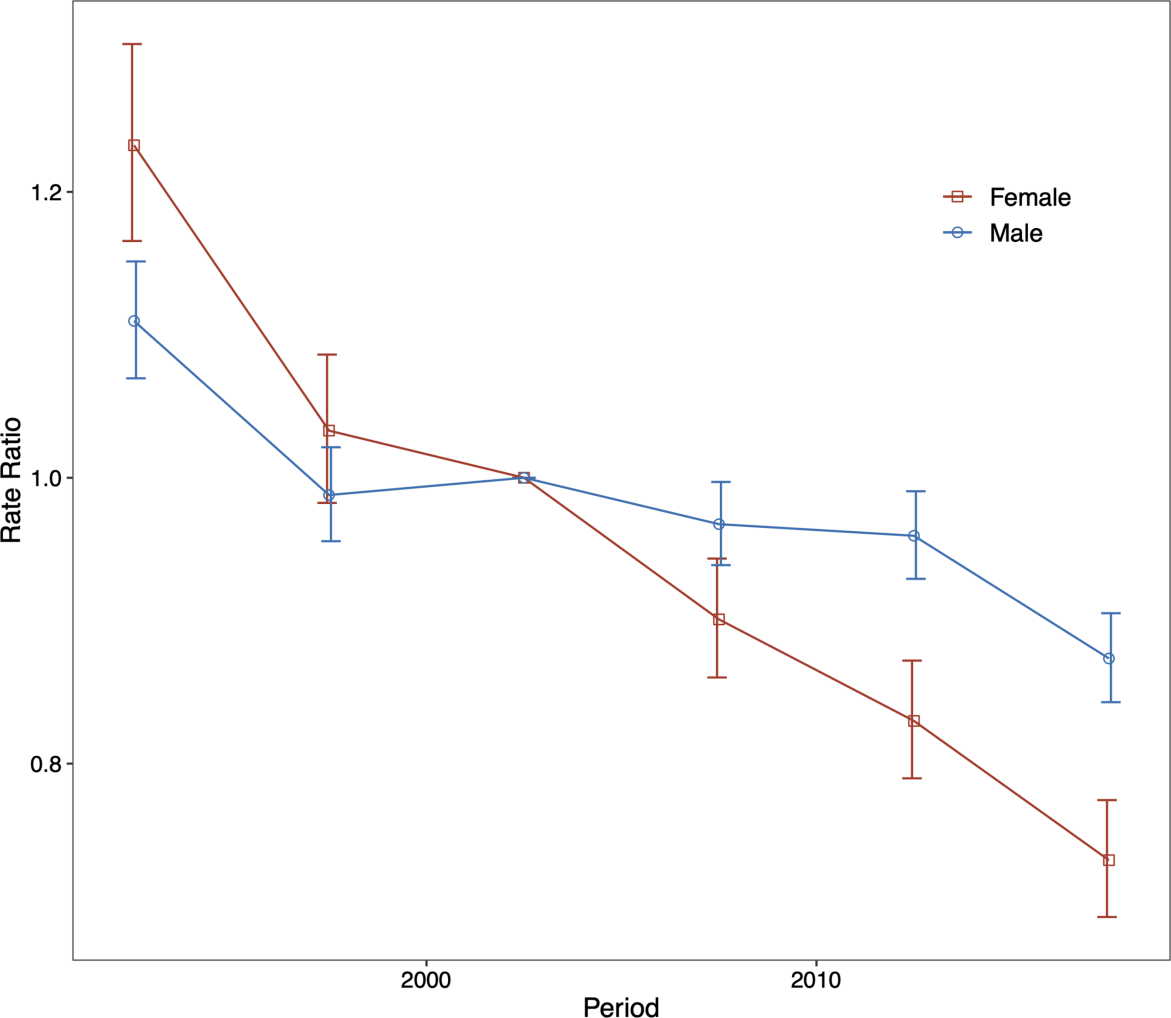
Relative risk of each period compared with the reference period (2000–2004) adjusted for age and nonlinear cohort effects and the corresponding 95% confidence interval for males and females.

**Figure 5:**
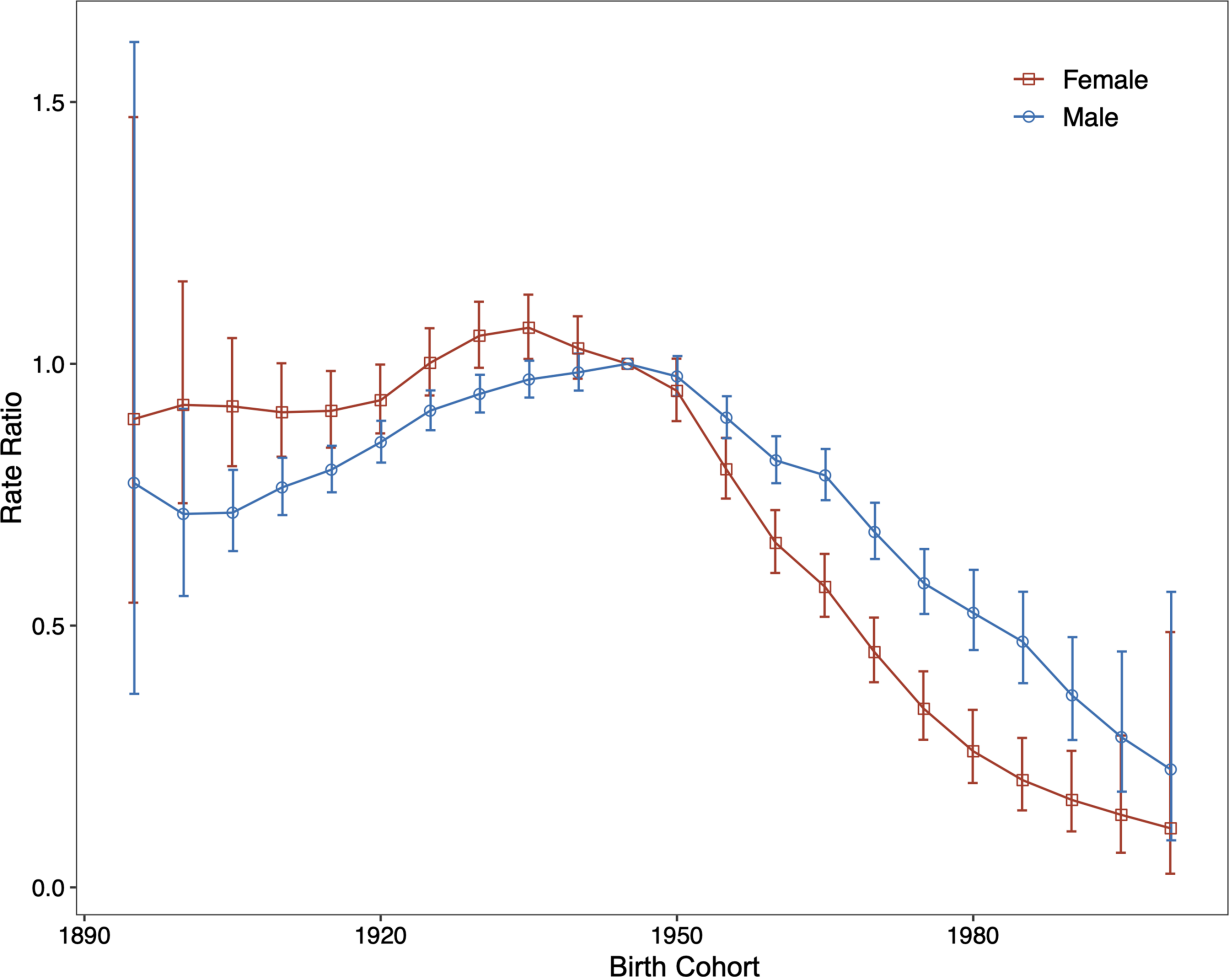
Relative risk of each cohort compared with the reference cohort (cohort 1945–1949) adjusted for age and nonlinear period effects and the corresponding 95% confidence interval.

The period (cohort) relative risks are the ratio of age-specific rates in each period (cohort) relative to the reference period (cohort). We found decreased period relative risks for both sexes, with a more quickly decreasing trend for females than for males during the whole study period after adjusting for age and birth cohort (**Figure 4**). Cohort relative risks were also found in similar patterns for both sexes, starting to decline after 1935 for females and after 1945 for males and then declining more rapidly for females (**Figure 5**). In addition, using the specific results of Wald tests, we found cohort and period effects for both sexes, and the net drifts and local drifts were all statistically significant (*p*<0.05) (**Table S1**).

### DKD Mortality Projection

We next conducted a Bayesian age-period-cohort analysis to project future mortality trends for DKD in China. Our results showed that the total number of deaths from DKD in China would continue to increase, with 88,803 deaths from DKD in China by 2030 (**Table S2**). However, there were significant differences in the distribution of DKD deaths across age groups, with more occurring in the older age groups and a continued increase in the older age groups (above 60 years), but a decreasing trend in the younger age groups (under 50 years).

### Decomposition Analysis

**Figure 6:**
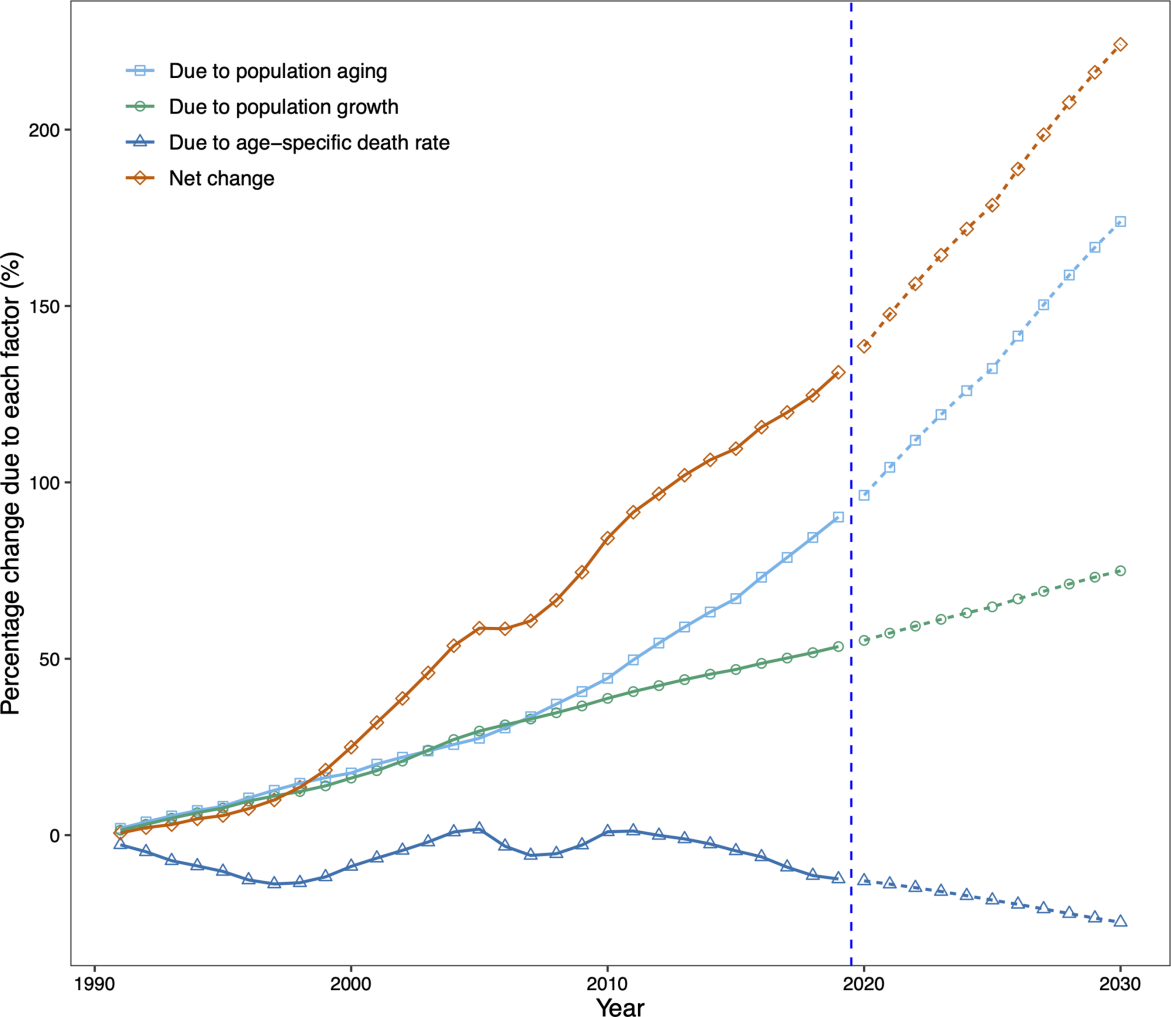
Contribution of changes in population aging, population growth, and age-specific type 2 diabetic kidney disease (DKD) death rate to changes in number of DKD deaths from 1991 to 2030 for both sexes in China, using 1990 as the reference year. Data in the right of the blue dashed line were the decomposition based on the projected data. DKD, type 2 diabetic kidney disease.

Finally, we conducted a demographic decomposition analysis to identify DKD mortality drivers from 1990 to 2030. Our results showed that demographic factors drove the increasing trend in the number of DKD deaths in China, with population aging playing a dominant role, especially after 2010 (**Table 1**, **Figure 6**).

**Table 1:** Contribution of changes in population aging, population growth, and age-specific death rate of type 2 diabetic kidney disease (DKD) to the net change of DKD deaths in China from 1991 to 2030, using 1990 as the reference year.

Year	Due to population aging, n (%)	Due to population growth, n (%)	Due to age-specific death rate, n (%)	Net change n (%)
1991	526 (1.9)	386 (1.4)	-748 (-2.7)	164 (0.6)
1992	1030 (3.8)	842 (3.1)	-1291 (-4.7)	581 (2.1)
1993	1493 (5.4)	1314 (4.8)	-1987 (-7.3)	820 (3.0)
1994	1913 (7.0)	1745 (6.4)	-2390 (-8.7)	1267 (4.6)
1995	2237 (8.2)	2102 (7.7)	-2818 (-10.3)	1521 (5.6)
1996	2889 (10.5)	2646 (9.7)	-3480 (-12.7)	2055 (7.5)
1997	3483 (12.7)	3040 (11.1)	-3792 (-13.8)	2732 (10.0)
1998	4026 (14.7)	3387 (12.4)	-3696 (-13.5)	3716 (13.6)
1999	4462 (16.3)	3827 (14.0)	-3240 (-11.8)	5049 (18.4)
2000	4827 (17.6)	4430 (16.2)	-2428 (-8.9)	6829 (24.9)
2001	5519 (20.1)	5008 (18.3)	-1785 (-6.5)	8741 (31.9)
2002	6049 (22.1)	5749 (21.0)	-1183 (-4.3)	10615 (38.8)
2003	6536 (23.9)	6596 (24.1)	-534 (-1.9)	12598 (46.0)
2004	7037 (25.7)	7425 (27.1)	244 (0.9)	14707 (53.7)
2005	7521 (27.5)	8083 (29.5)	467 (1.7)	16071 (58.7)
2006	8325 (30.4)	8563 (31.3)	-863 (-3.2)	16025 (58.5)
2007	9197 (33.6)	9017 (32.9)	-1575 (-5.7)	16639 (60.7)
2008	10175 (37.1)	9502 (34.7)	-1441 (-5.3)	18236 (66.6)
2009	11150 (40.7)	10033 (36.6)	-768 (-2.8)	20416 (74.5)
2010	12178 (44.5)	10621 (38.8)	260 (1.0)	23059 (84.2)
2011	13608 (49.7)	11148 (40.7)	319 (1.2)	25075 (91.5)
2012	14914 (54.4)	11612 (42.4)	-21 (-0.1)	26505 (96.8)
2013	16162 (59.0)	12077 (44.1)	-292 (-1.1)	27947 (102.0)
2014	17329 (63.3)	12498 (45.6)	-686 (-2.5)	29140 (106.4)
2015	18367 (67.0)	12862 (47.0)	-1219 (-4.5)	30010 (109.6)
2016	20027 (73.1)	13338 (48.7)	-1685 (-6.2)	31680 (115.6)
2017	21560 (78.7)	13749 (50.2)	-2485 (-9.1)	32825 (119.8)
2018	23112 (84.4)	14173 (51.7)	-3138 (-11.5)	34147 (124.7)
2019	24701 (90.2)	14644 (53.5)	-3403 (-12.4)	35942 (131.2)
2020	26392 (96.3)	15121 (55.2)	-3553 (-13.0)	37960 (138.6)
2021	28563 (104.3)	15684 (57.3)	-3802 (-13.9)	40446 (147.6)
2022	30661 (111.9)	16230 (59.2)	-4079 (-14.9)	42812 (156.3)
2023	32655 (119.2)	16755 (61.2)	-4376 (-16.0)	45034 (164.4)
2024	34514 (126.0)	17254 (63.0)	-4702 (-17.2)	47066 (171.8)
2025	36237 (132.3)	17725 (64.7)	-5045 (-18.4)	48917 (178.6)
2026	38754 (141.5)	18342 (67.0)	-5371 (-19.6)	51725 (188.8)
2027	41185 (150.3)	18934 (69.1)	-5729 (-20.9)	54390 (198.6)
2028	43493 (158.8)	19498 (71.2)	-6090 (-22.2)	56901 (207.7)
2029	45648 (166.6)	20030 (73.1)	-6442 (-23.5)	59236 (216.2)
2030	47650 (173.9)	20528 (74.9)	-6768 (-24.7)	61410 (224.2)

DKD, type 2 diabetic kidney disease.

There were 35,942 additional DKD deaths in China in 2019 from 1990, an increase of 131.2%. The increase was driven by changes in the number of DKD deaths due to population aging (90.2% increase from 1990) and population growth (53.5% increase from 1990). Our projection suggests that the increasing trend in the number of DKD deaths will continue. By 2030, China will have 224.2% more DKD deaths than in 1990, with a contribution of 173.9% increase in deaths due to population aging and a 74.9% increase due to population growth, despite a 24.7% decrease in age-specific death rates (**Table 1**, **Figure 6**).

## Discussion

In this study, we estimated age, period, and cohort effects in DKD mortality between 1990 and 2019 using age-period-cohort analyses, predicted DKD deaths from 2020 to 2030 using Bayesian age-period-cohort analysis with integrated nested Laplace approximations and decomposed the main drivers of the changes in DKD deaths from 1990 to 2030. We found a decreasing trend in both cohort and period effects for DKD deaths in China, suggesting the success of earlier policies in reducing DKD deaths. We estimated that by 2030, DKD deaths would increase dramatically by 224.2% from 1990, driven primarily by population aging, which completely offset the reduction in DKD deaths due to epidemiological changes.

Although previous studies have shown an association between DKD mortality and age (1, 8), we quantitatively demonstrated an exponential increase in DKD mortality with age after adjusting for period and cohort effects. This age effect may be partly due to the more unsatisfactory treatment outcome and prognosis of DKD with increasing age. At the same time, the higher all-cause mortality may explain the higher DKD morality for males than for females (31).

Improvements in medical conditions are the main reason for the monotonic decline between the period and DKD mortality. Fast urbanization and advances in primary health care in China over the last three decades have promoted the availability, accessibility, and affordability of health care services, especially as the Chinese government has continued to improve the health care system in recent years, resulting in more than 99.9% of the poor population participating in basic health insurance, these initiatives have greatly improved the treatment of DKD, for instance, the affordability and accessibility of dialysis have been greatly improved (11, 32), thus significantly reducing the mortality of DKD.

The decline in the cohort effects of DKD mortality may be due to improved medical conditions, with more deaths due to DKD among those born before the 1950s and a gradual downward trend in DKD deaths in the post-1950 cohort. The lack of nutritional conditions in early life may be a risk factor for the high incidence of diabetes and kidney disease in adulthood (33). At the same time, social unrest in China before 1950 may have contributed to nutritional deficiencies in early life. In addition, better education and better awareness of diabetes in successive generations may have played a partial role (34). It is worth pointing out that although the period and cohort effects can be estimated as period relative risk and cohort relative risk, respectively, it is not appropriate to interpret them completely separately (12, 13, 35), because there is an interaction between the two.

Our study showed that the number of DKD deaths in China had increased significantly over the past three decades. In contrast, the age-standardized DKD mortality rate has fluctuated only marginally. The inconsistency reflects the vital role that demographic change plays in DKD deaths. While significant improvements in DKD diagnosis, treatment, and management techniques in recent decades, accompanied by more and better healthcare professionals, have played a key role in reducing deaths from DKD, however, these advances have been offset by changes in demographics and population size. Population aging has become the main dominant driver in the absolute number of DKD deaths in China, and this trend is set to continue as the population continues to age. In contrast, the role of population growth is relatively weak. These suggest that China needs to allocate healthcare resources to cope with the changes in healthcare needs brought about by an aging population.

It is noteworthy that the prevalence of risk factors for DKD in China, particularly diabetes, is not well controlled. For example, the prevalence of diabetes (a major DKD risk factor) has increased rapidly in the Chinese adult population since 1990 (36). However, we also note that effective and increasingly common measures to control blood glucose and large-scale screening for chronic kidney disease may alleviate the burden of diabetes to some extent and further alleviate the burden of DKD (7, 37).

The present study has some limitations. First, the Bayesian age-period-cohort requires fixed age and period intervals, however the age group of 95 and plus recorded in the GBD 2019 database might vary by years. We expect the varying 95 and plus age interval should not materially affect our results given the proportion of people aged over 100 years is small. Second, GBD 2019 includes limited sources from a small number of countries (8, 18), and only used data from the end-stage renal registry to model the proportion of deaths due to CKD, without considering other causes of DKD deaths such as nephrotic syndrome, so there is likely to be some uncertainty in the DKD estimates in China (18). Third, there may be some uncertainty in the UN projections of the size and distribution of China’s population, which could affect the population-based analysis, such as decomposition and projection. Fourth, we contributed the increase in DKD deaths into population growth, population aging, and epidemiologic changes and did not further examine other factors that could influence DKD deaths, such as age at onset age of diabetes and DKD, diabetic duration, and blood control, due to the lack of available data in the GBD database.

## Conclusion

The burden of DKD deaths in China is likely to continue increasing. Although China has made progress in reducing DKD deaths, demographic changes have entirely offset the progress, primarily driven by population aging. Our findings suggest the urgency of improving health systems to meet the health needs of older adults, and the importance of large-scale screening and risk factor control for DKD control and prevention.

## Data Availability Statement

The original contributions presented in the study are included in the article/**Supplementary Material**. Further inquiries can be directed to the corresponding author.

## Author Contributions

XW, JD, and SS designed the study, wrote, reviewed, and edited the manuscript. LL and WC reviewed and contributed to edit the manuscript. XW, JD, and LL researched and analyzed data. JD is the guarantor of this work. All authors contributed to the article and approved the submitted version.

## Funding

This research was supported by Science and Technology Project of Xi’an City, China (No. 2019GXYD11.2).

## Conflict of Interest

The authors declare that the research was conducted in the absence of any commercial or financial relationships that could be construed as a potential conflict of interest.

## Publisher’s Note

All claims expressed in this article are solely those of the authors and do not necessarily represent those of their affiliated organizations, or those of the publisher, the editors and the reviewers. Any product that may be evaluated in this article, or claim that may be made by its manufacturer, is not guaranteed or endorsed by the publisher.

## References

[1] Fineberg D Jandeleit-Dahm KA Cooper ME. Diabetic Nephropathy: Diagnosis and Treatment. Nat Rev Endocrinol (2013) 9:713. doi: 10.1038/nrendo.2013.184 24100266

[2] Thomas MC Brownlee M Susztak K Sharma K Jandeleit-Dahm KA Zoungas S. Diabetic Kidney Disease. Nat Rev Dis Primers (2015) 1:1–20. doi: 10.1038/nrdp.2015.18 PMC7724636 27188921

[3] Thomas MC Cooper ME Zimmet P. Changing Epidemiology of Type 2 Diabetes Mellitus and Associated Chronic Kidney Disease. Nat Rev Nephrol (2016) 12:73. doi: 10.1038/nrneph.2015.173 26553517

[4] Afkarian M Zelnick LR Hall YN Heagerty PJ Tuttle K Weiss NS. Clinical Manifestations of Kidney Disease Among US Adults With Diabetes, 1988-2014. JAMA (2016) 316:602–10. doi: 10.1001/jama.2016.10924 PMC5444809 27532915

[5] Alicic RZ Rooney MT Tuttle KR. Diabetic Kidney Disease: Challenges, Progress, and Possibilities. Clin J Am Soc Nephrol (2017) 12:2032–45. doi: 10.2215/CJN.11491116 PMC5718284 28522654

[6] Giorda CB Carnà P Salomone M Picariello R Costa G Tartaglino B. Ten-Year Comparative Analysis of Incidence, Prognosis, and Associated Factors for Dialysis and Renal Transplantation in Type 1 and Type 2 Diabetes Versus Non-Diabetes. Acta Diabetol (2018) 55:733–40. doi: 10.1007/s00592-018-1142-y 29679150

[7] Zhang L Long J Jiang W Shi Y He X Zhou Z. Trends in Chronic Kidney Disease in China. N Engl J Med (2016) 375:905–6. doi: 10.1056/NEJMc1602469 27579659

[8] de Boer IH Rue TC Hall YN Heagerty PJ Weiss NS Himmelfarb J. Temporal Trends in the Prevalence of Diabetic Kidney Disease in the United States. JAMA (2011) 305:2532–9. doi: 10.1001/jama.2011.861 PMC3731378 21693741

[9] Liu L Gao B Wang J Yang C Wu S Wu Y. Time-Averaged Serum Uric Acid and 10-Year Incident Diabetic Kidney Disease: A Prospective Study From China. J Diabetes (2020) 12:169–78. doi: 10.1111/1753-0407.12983 31461212

[10] Zhang XX Kong J Yun K. Prevalence of Diabetic Nephropathy Among Patients With Type 2 Diabetes Mellitus in China: A Meta-Analysis of Observational Studies. J Diabetes Res (2020) 2020:2315607. doi: 10.1155/2020/2315607 32090116 PMC7023800

[11] Zhang Z Cui T Cui M Kong X. High Prevalence of Chronic Kidney Disease Among Patients With Diabetic Foot: A Cross-Sectional Study at a Tertiary Hospital in China. Nephrol (Carlton) (2020) 25:150–5. doi: 10.1111/nep.13596 31025471

[12] Zou Z Cini K Dong B Ma Y Ma J Burgner DP. Time Trends in Cardiovascular Disease Mortality Across the BRICS: An Age-Period-Cohort Analysis of Key Nations With Emerging Economies Using the Global Burden of Disease Study 2017. Circulation (2020) 141:790–9. doi: 10.1161/CIRCULATIONAHA.119.042864 31941371

[13] Jacobs D Huang H Olino K Weiss S Kluger H Judson BL. Assessment of Age, Period, and Birth Cohort Effects and Trends in Merkel Cell Carcinoma Incidence in the United States. JAMA Dermatol (2021) 157:59–65. doi: 10.1001/jamadermatol.2020.4102 33146688 PMC7643047

[14] Bray F Møller B. Predicting the Future Burden of Cancer. Nat Rev Cancer (2006) 6:63–74. doi: 10.1038/nrc1781 16372017

[15] Chen WQ Zheng RS Zeng HM. Bayesian Age-Period-Cohort Prediction of Lung Cancer Incidence in China. Thorac Cancer (2011) 2:149–55. doi: 10.1111/j.1759-7714.2011.00062.x 27755842

[16] Cheng X Tan L Gao Y Yang Y Schwebel DC Hu G. A New Method to Attribute Differences in Total Deaths Between Groups to Population Size, Age Structure and Age-Specific Mortality Rate. PloS One (2019) 14:e0216613. doi: 10.1371/journal.pone.0216613 31075117 PMC6510436

[17] Vos T Lim SS Abbafati C Abbas KM Abbasi M Abbasifard M. Global Burden of 369 Diseases and Injuries in 204 Countries and Territories, 1990–2019: A Systematic Analysis for the Global Burden of Disease Study 2019. Lancet (2020) 396:1204–22. doi: 10.1016/S0140-6736(20)30925-9 PMC7567026 33069326

[18] Murray CJ Aravkin AY Zheng P Abbafati C Abbas KM Abbasi-Kangevari M. Global Burden of 87 Risk Factors in 204 Countries and Territories, 1990–2019: A Systematic Analysis for the Global Burden of Disease Study 2019. Lancet (2020) 396:1223–49. doi: 10.1016/S0140-6736(20)30752-2 PMC7566194 33069327

[19] Bikbov B Purcell CA Levey AS Smith M Abdoli A Abebe M. Global, Regional, and National Burden of Chronic Kidney Disease, 1990–2017: A Systematic Analysis for the Global Burden of Disease Study 2017. Lancet (2020) 395:709–33. doi: 10.1016/S0140-6736(20)30045-3 PMC7049905 32061315

[20] Rosenberg PS Check DP Anderson WF. A Web Tool for Age–Period–Cohort Analysis of Cancer Incidence and Mortality Rates. Cancer Epidemiol Biomarkers Prev (2014) 23:2296–302. doi: 10.1158/1055-9965.EPI-14-0300 PMC4221491 25146089

[21] Riebler A Held L. Projecting the Future Burden of Cancer: Bayesian Age–Period–Cohort Analysis With Integrated Nested Laplace Approximations. Biom J (2017) 59:531–49. doi: 10.1002/bimj.201500263 28139001

[22] Knoll M Furkel J Debus J Abdollahi A Karch A Stock C. An R Package for an Integrated Evaluation of Statistical Approaches to Cancer Incidence Projection. BMC Med Res Methodol (2020) 20:1–11. doi: 10.1186/s12874-020-01133-5 PMC7559591 33059585

[23] Desa U. World Population Prospects 2019. In: World Population Prospects 2019. United Nations: Department of Economic and Social Affairs (2019).

[24] Cheng X Yang Y Schwebel DC Liu Z Li L Cheng P. Population Ageing and Mortality During 1990–2017: A Global Decomposition Analysis. PloS Med (2020) 17:e1003138. doi: 10.1371/journal.pmed.1003138 32511229 PMC7279585

[25] Roth GA Abate D Abate KH Abay SM Abbafati C Abbasi N. Global, Regional, and National Age-Sex-Specific Mortality for 282 Causes of Death in 195 Countries and Territories, 1980–2017: A Systematic Analysis for the Global Burden of Disease Study 2017. Lancet (2018) 392:1736–88. doi: 10.1016/S0140-6736(18)32203-7 PMC6227606 30496103

[26] Burden G Fitzmaurice C Akinyemiju T Al Lami F Alam T Alizadeh-Navaei R. Global, Regional, and National Cancer Incidence, Mortality, Years of Life Lost, Years Lived With Disability, and Disability-Adjusted Life-Years for 29 Cancer Groups, 1990 to 2016: A Systematic Analysis for the Global Burden of Disease Study. JAMA Oncol (2018) 4:1553–68. doi: 10.1001/jamaoncol.2018.2706 PMC6248091 29860482

[27] Wang L Wu X Du J Cao W Sun S. Global Burden of Ischemic Heart Disease Attributable to Ambient PM2.5 Pollution From 1990 to 2017. Chemosphere (2021) 263:128134. doi: 10.1016/j.chemosphere.2020.128134 33297122

[28] Du J Yang J Wang L Wu X Cao W Sun S. A Comparative Study of the Disease Burden Attributable to PM2.5 in China, Japan and South Korea From 1990 to 2017. Ecotoxicol Environ (2021) 209:111856. doi: 10.1016/j.ecoenv.2020.111856 33412383

[29] Wang L Du J Cao W Sun S. Trends of Stroke Attributable to High Sodium Intake at the Global, Regional, and National Levels From 1990 to 2019: A Population-Based Study. Neurol Res (2021) 43:474–81. doi: 10.1080/01616412.2020.1867950 33377423

[30] Roth GA Forouzanfar MH Moran AE Barber R Nguyen G Feigin VL. Demographic and Epidemiologic Drivers of Global Cardiovascular Mortality. N Engl J Med (2015) 372:1333–41. doi: 10.1056/NEJMoa1406656 PMC4482354 25830423

[31] Wang H Naghavi M Allen C Barber RM Bhutta ZA Carter A. Global, Regional, and National Life Expectancy, All-Cause Mortality, and Cause-Specific Mortality for 249 Causes of Death, 1980–2015: A Systematic Analysis for the Global Burden of Disease Study 2015. Lancet (2016) 388:1459–544. doi: 10.1016/S0140-6736(16)31012-1 PMC5388903 27733281

[32] Gao B Wu S Wang J Yang C Chen S Hou J. Clinical Features and Long-Term Outcomes of Diabetic Kidney Disease–A Prospective Cohort Study From China. J Diabetes Complicat (2019) 33:39–45. doi: 10.1016/j.jdiacomp.2018.09.019 30482493

[33] Roseboom TJ Painter RC van Abeelen AF Veenendaal MV de Rooij SR. Hungry in the Womb: What Are the Consequences? Lessons From the Dutch Famine. Maturitas (2011) 70:141–5. doi: 10.1016/j.maturitas.2011.06.017 21802226

[34] Whaley-Connell A Shlipak MG Inker LA Tamura MK Bomback AS Saab G. Awareness of Kidney Disease and Relationship to End-Stage Renal Disease and Mortality. Am J Med (2012) 125:661–9. doi: 10.1016/j.amjmed.2011.11.026 PMC3383388 22626510

[35] Wang Z Hu S Sang S Luo L Yu C. Age-Period-Cohort Analysis of Stroke Mortality in China: Data From the Global Burden of Disease Study 2013. Stroke (2017) 48:271–5. doi: 10.1161/STROKEAHA.116.015031 27965429

[36] Liu X Yu C Wang Y Bi Y Liu Y Zhang Z-J. Trends in the Incidence and Mortality of Diabetes in China From 1990 to 2017: A Joinpoint and Age-Period-Cohort Analysis. Int J Environ Res Public Health (2019) 16:158. doi: 10.3390/ijerph16010158 PMC6339039 30626127

[37] Go DS Kim SH Park J Ryu DR Lee HJ Jo MW. Cost-Utility Analysis of the National Health Screening Program for Chronic Kidney Disease in Korea. Nephrology (2019) 24:56–64. doi: 10.1111/nep.13203 29206319

