# Supplementary material for: Bayesian Age-Period-Cohort Prediction of Mortality of Type 2 Diabetic Kidney Disease in China: A Modeling Study
Source: Front Endocrinol (Lausanne). 2021 Oct 29;12:767263. doi: 10.3389/fendo.2021.767263 (PMC8586507; doi:10.3389/fendo.2021.767263)
Supplement: Supplementary file 1 [file Table_1.docx]

**Supplementary Material**

**Bayesian age-period-cohort prediction of mortality of type 2 diabetic kidney disease**

**in China: A modeling study**

Jianqiang Du, Xiangbo Chen, Linchang Li, Xiaoming Wu, Wangnan Cao, Shengzhi Sun

**Table of content**

The decomposition method

Projection of future mortality

Table S1. Wald Chi-square tests for estimable parameters in the age-period-cohort model

Table S2. Estimated number of age-specific type 2 diabetic kidney disease (DKD) deaths in China from 2020 to 2030

**The decomposition method**

The population decomposition algorithm has been described in detail in the papers by Cheng et. al [1,2]. Briefly, take the difference in the number of DKD deaths between 1990 and 2019 in China, we can decompose the net change of death into the contribution of three factors, namely, population growth, population aging, and age-specific death rate.

The age groups were divided using 5-year increments from 15-19 years to 95 plus (we included older people aged ≥95 years as the 95-99 years age group, which was recorded as only one group in the GBD 2019). Let *d_ij_*, *n_ij_*, *m_ij_* and *s_ij_* denote the number of death, population size, age-specific rate of death, and population proportion in the *i*^th^ age group of the year *j*, respectively, (*i* = 1, 2, …, 20; *j* = 1, 2). Let *D*_1_ and *D*_2_, *N*_1_ and *N*_2_, *P*_1_ and *P*_2_ represent the total number of death, population size, and the crude rate of death in 1990 and 2019, respectively.

Using *M_p_*, *M_a_* and *M_m_* to represent the main effects of the changes in population size, age structure and death rate, and *I_pa_*, *I_pm_*, *I_am_* and *I_pam_* to represent their two-way and three-way interactions, respectively. In the case of 1990 as the reference year, these terms are calculated as follows:

$M_{p}=\sum_{i=1}^{20} {{\left( N_{2}-N_{1} \right)s}_{i1}m}_{i1}$

$M_{a}=\sum_{i=1}^{20} N_{1}\left( s_{i2}-s_{i1} \right)m_{i1}$

$M_{m}=\sum_{i=1}^{20} {N_{1}s}_{i1}\left( m_{i2}-m_{i1} \right)$

$I_{pa}=\sum_{i=1}^{20} \left( N_{2}-N_{1} \right)\left( s_{i2}-s_{i1} \right)m_{i1}$

$I_{pm}=\sum_{i=1}^{20} \left( N_{2}-N_{1} \right)s_{i1}\left( m_{i2}-m_{i1} \right)$

$I_{am}=\sum_{i=1}^{20} N_{1}\left( s_{i2}-s_{i1} \right)\left( m_{i2}-m_{i1} \right)$

$I_{pam}=\sum_{i=1}^{20} \left( N_{2}-N_{1} \right)\left( s_{i2}-s_{i1} \right)\left( m_{i2}-m_{i1} \right)$

Here, a simplification needs to be made, assuming that the interactions are equally distributed, then the contribution of the three factors can be calculated as follows:

$A{=M}_{a}+½I_{am}+½I_{pa}+⅓I_{pam}$

$P{=M}_{p}+½I_{pm}+½I_{pa}+⅓I_{pam}$

$M{=M}_{m}+½I_{pm}+½I_{am}+⅓I_{pam}$

Net change = *D_2_ - D_1_*

Here, *A* represents the contribution of population aging, *P* represents the contribution of population growth, *M* represents the contribution of the age-specific death rate, and net change represents total change. The contribution of the each factor divided by *D_1_* and multiplied by 100 is the percentage of the respective contribution.

**References**

1. Cheng X, Yang Y, Schwebel DC, Liu Z, Li L, Cheng P et al. Population ageing and mortality during 1990-2017: A global decomposition analysis. PLoS Med 2020;17:e1003138.

2. Cheng X, Tan L, Gao Y, Yang Y, Schwebel DC , Hu G. A new method to attribute differences in total deaths between groups to population size, age structure and age-specific mortality rate. PLoS One 2019;14:e0216613.

**Projection of future mortality**

The future mortality was projected using the Bayesian age-period-cohort analysis with integrated nested Laplace approximations (INLA). The Bayesian approach attributes separate effects to age, period and cohort, and extrapolates these effects to make projections, it does not depend on strong parametric assumptions like the classical approach, it is the only current method to achieve nonarbitrary and sensible projections.

Based on the expectation that effects adjacent in time might be similar, the Bayesian inference in age-period-cohort model applies the second-order random walk for smoothing priors of age, period, and cohort effects and to project posterior mortality rates. According to this model, each point of effects is predicted by linear extrapolation from its two immediate predecessors, plus a random variance from a normal distribution with mean zero. The INLA is used with this Bayesian age-period-cohort model to approximate the marginal posterior distributions avoiding any mixing and convergence issues introduced by Markov chain Monte Carlo sampling techniques traditionally used in the Bayesian approach. The Bayesian age-period-cohort analysis was conducted by a R-package BAPC (version 0.0.34).

We prepared age-specific DKD mortality data (from 1990 to 2019) and Chinese population data (from 1990 to 2030), followed by an 11-year (from 2020 to 2030) retrospective projection using BAPC function in BAPC package.

**References**

1. Riebler, A. and L. Held, Projecting the future burden of cancer: Bayesian age-period-cohort analysis with integrated nested Laplace approximations. Biometrical Journal, 2017. 59(3): p. 531-549.

2. Jacobs, D., et al., Assessment of Age, Period, and Birth Cohort Effects and Trends in Merkel Cell Carcinoma Incidence in the United States. Jama Dermatology, 2021. 157(1): p. 59-65.

**Table S1. Wald Chi-squre tests for estimable parameters in the age-period-cohort model.** RR=relative risk.

| Null Hypothesis | Males | | Females | |
| --- | --- | --- | --- | --- |
|  | Chi-squre | P-value | Chi-squre | P-value |
| NetDrift = 0 | 65.8 | <0.001 | 169.7 | <0.001 |
| All Age Deviations = 0 | 459.6 | <0.001 | 45.6 | <0.001 |
| All Period RR = 1 | 112.2 | <0.001 | 185.1 | <0.001 |
| All Cohort RR = 1 | 371.7 | <0.001 | 375.6 | <0.001 |
| All Local Drifts = Net Drift | 363.2 | <0.001 | 343. 8 | <0.001 |

**Table S2. Estimated number of age-specific type 2 diabetic kidney disease** (**DKD) deaths in China from 2020 to 2030.** The data were projected by the bayesian age-period-cohort method.

| Year | Number of age-specific and total DKD deaths | | | | | | | | | | | | | | | | | |
| --- | --- | --- | --- | --- | --- | --- | --- | --- | --- | --- | --- | --- | --- | --- | --- | --- | --- | --- |
|  | 15-19 | 20-24 | 25-29 | 30-34 | 35-39 | 40-44 | 45-49 | 50-54 | 55-59 | 60-64 | 65-69 | 70-74 | 75-79 | 80-84 | 85-89 | 90-94 | 95 plus | Total |
| 2020 | 20 | 48 | 99 | 249 | 355 | 611 | 1352 | 2787 | 3917 | 5008 | 8448 | 10019 | 10618 | 10309 | 7905 | 2867 | 741 | 65354 |
| 2021 | 19 | 45 | 90 | 235 | 362 | 584 | 1266 | 2712 | 4041 | 5033 | 8323 | 10675 | 11129 | 10601 | 8514 | 3346 | 864 | 67839 |
| 2022 | 18 | 42 | 84 | 215 | 374 | 565 | 1175 | 2610 | 4152 | 5159 | 8034 | 11461 | 11663 | 10848 | 9040 | 3722 | 1043 | 70205 |
| 2023 | 17 | 40 | 79 | 191 | 385 | 554 | 1088 | 2491 | 4232 | 5361 | 7694 | 12228 | 12275 | 11131 | 9433 | 4022 | 1206 | 72428 |
| 2024 | 17 | 37 | 74 | 170 | 387 | 551 | 1014 | 2363 | 4253 | 5597 | 7458 | 12754 | 13060 | 11537 | 9700 | 4200 | 1287 | 74460 |
| 2025 | 16 | 36 | 70 | 153 | 376 | 556 | 958 | 2228 | 4204 | 5827 | 7407 | 12954 | 14060 | 12109 | 9863 | 4262 | 1231 | 76311 |
| 2026 | 16 | 34 | 66 | 140 | 356 | 568 | 918 | 2089 | 4098 | 6022 | 7469 | 12776 | 15141 | 12841 | 10348 | 4813 | 1424 | 79119 |
| 2027 | 15 | 33 | 62 | 130 | 326 | 588 | 890 | 1944 | 3954 | 6206 | 7680 | 12359 | 16405 | 13541 | 10738 | 5216 | 1697 | 81784 |
| 2028 | 15 | 32 | 58 | 123 | 291 | 607 | 875 | 1805 | 3786 | 6348 | 8008 | 11892 | 17607 | 14323 | 11100 | 5495 | 1931 | 84295 |
| 2029 | 14 | 30 | 55 | 116 | 260 | 612 | 873 | 1689 | 3604 | 6404 | 8394 | 11612 | 18425 | 15352 | 11540 | 5618 | 2030 | 86630 |
| 2030 | 14 | 30 | 53 | 110 | 235 | 597 | 884 | 1602 | 3413 | 6360 | 8788 | 11635 | 18756 | 16690 | 12128 | 5582 | 1927 | 88803 |
